# Growth Performance, Milk Productivity, Productive Longevity, and Milk Composition of Mugalzhar Horses

**DOI:** 10.3390/ani16142272

**Published:** 2026-07-22

**Authors:** Maxat Toishimanov, Oralbek Alikhanov, Khamit Aubakirov, Dilaram Karibayeva, Makpal Kargaeva, Zagipa Sapakhova, Dinara Begaliyeva, Rakhim Kanat, Yuliya Tugunova, Dias Daurov, Ainash Daurova, Kabyl Zhambakin, Malika Shamekova, Dastanbek Baimukanov

**Affiliations:** 1 Laboratory of Breeding and Biotechnology, Institute of Plant Biology and Biotechnology, Timiryazev 45, Almaty 050040, Kazakhstan; 2 Research Laboratory Agricultural Biotechnology, Department of Biotechnology, Auezov South Kazakhstan University, Tauke-Khan Avenue 5, Shymkent 160012, Kazakhstan; 3 Zhambyl Branch of the Kazakh Scientific Research Veterinary Institute, 87 Abai Avenue, Taraz 080003, Kazakhstan; 4 Testing Center, Zhangir Khan West-Kazakhstan Agrarian Technical University, Zhangir Khan 51, Oral 090009, Kazakhstan; 5 Department of Animal Husbandry, Veterinary Medicine and Assessment of Feed and Milk Quality, Research and Production Center for Animal Husbandry and Veterinary Medicine, Astana 010000, Kazakhstan; 6 Tanir Research Laboratory, Al-Farabi Avenue 75B, Almaty 050060, Kazakhstan

**Keywords:** mare’s milk, lifetime productivity, functional longevity, lactation performance, phenotypic characterization, principal component analysis, productive herd life, genetic resources

## Abstract

The Mugalzhar horse is an important native breed of Kazakhstan, valued for its adaptability, milk production, and meat productivity under extensive pasture-based management. This study evaluated the relationships among first-lactation milk yield, productive longevity, lifetime milk production, growth, morphometric traits, and milk composition in the Mugalzhar horse breed using data from 100 mares with complete lifetime production records, 108 adult mares and 12 stallions for morphometric evaluation, and 68 foals (35 colts and 33 fillies) for growth analysis. The study was conducted on Mugalzhar horses maintained at the “Senim” farm in the Turkestan region of Kazakhstan. Morphometric measurements of adult horses, growth dynamics of foals from birth to 18 months of age, milk productivity records, productive longevity indicators, and milk composition data were analyzed. PCA, Pearson correlation analysis, and HCA were applied to assess relationships among productive and compositional traits. Adult stallions and mares closely conformed to breed standards, with stallions exceeding mares by approximately 7.8% in live weight. Foals exhibited intensive postnatal growth, with live weight increasing 6.8–6.9-fold between birth and 18 months of age. Milk yield increased from 722.3 kg during the first lactation to 986.9 kg during the fifth lactation before declining in later lactations, whereas milk composition remained relatively stable. Moderate-to-high first-lactation milk yield groups (G3–G4) achieved the greatest lifetime milk production (24,961–25,997 kg), longest productive life (7.9–8.5 years), and highest number of completed lactations (6.6–6.9). PCA revealed two major biological gradients representing productivity and longevity, while HCA identified G3–G4 as the most favorable production cluster. Milk fat and protein concentrations were negatively associated with milk yield traits. Moderate first-lactation milk yield was associated with the greatest lifetime productivity and longest productive herd life, whereas extremely high first-lactation milk yield was not associated with superior lifetime performance. These findings suggest that first-lactation milk yield may be considered together with longevity and other functional traits in future breeding programs for Mugalzhar horses; however, the results should be interpreted in light of the observational design of the present study.

## Simple Summary

The Mugalzhar horse is one of the most important native horse breeds of Kazakhstan and is widely used for the production of mare’s milk and meat under year-round pasture conditions. Understanding how growth, milk production, and productive lifespan are related is important for improving breeding efficiency and conserving this valuable genetic resource. In this study, we evaluated body measurements of adult horses, growth patterns of foals from birth to 18 months of age, milk production across lactations, and the relationship between first-lactation milk yield and lifetime productivity in Mugalzhar mares. The results showed that the studied horses closely matched breed standards and that foals exhibited rapid growth during the first months of life. Mares with moderate-to-high milk production during their first lactation achieved the greatest lifetime milk yield and remained productive for a longer period than mares with either very low or extremely high first-lactation milk yield. Milk yield increased up to the fifth lactation, whereas milk composition remained relatively stable. These findings indicate that selecting mares with balanced rather than maximum first-lactation milk yield may improve long-term productivity and herd profitability while supporting the sustainable conservation and utilization of the Mugalzhar horse breed.

## 1. Introduction

Horse breeding represents an important sector of livestock production in Kazakhstan, where horses are traditionally used for the production of meat and mare’s milk, the latter serving as the raw material for koumiss, a fermented dairy product of considerable nutritional and cultural importance. Among the native horse breeds of Kazakhstan, the Mugalzhar horse occupies a prominent position owing to its adaptability to year-round grazing, resistance to harsh environmental conditions, and dual-purpose productivity for both meat and milk production [1,2]. The breed was developed from the Jabe type of the Kazakh horse through long-term selection and was officially recognized as an independent breed in 1998 [3,4]. Today, the Mugalzhar horse is considered one of the most valuable genetic resources in Kazakhstan and is widely utilized in commercial horse-breeding systems [5].

Recent studies have demonstrated that the Mugalzhar breed possesses unique adaptive characteristics and considerable genetic diversity, reflecting its long-term adaptation to the continental climate of the Eurasian steppe [6,7]. In addition to genetic diversity, morphometric traits such as live weight, height at withers, body length, chest girth, and cannon girth are important indicators of breed development, productive potential, and conformity to breed standards [8,9]. Phenotypic characterization of local horse populations is therefore recognized as an essential component of breed conservation and sustainable breeding programs [10].

Mare’s milk has attracted increasing scientific interest because of its unique nutritional composition and functional properties. Compared with bovine milk, mare’s milk contains lower fat levels, higher lactose concentrations, a greater proportion of whey proteins, and a composition more closely resembling human milk [11,12]. In addition, mare’s milk contains numerous biologically active compounds, including lactoferrin, lysozyme, immunoglobulins, and bioactive peptides, which contribute to its nutritional and therapeutic value [13,14]. As a result, mare-milk products are consumed by millions of people worldwide, and demand for koumiss and other functional dairy products derived from mare’s milk continues to increase [15,16]. Consequently, improving milk productivity while maintaining milk quality has become an important objective in dairy horse breeding.

Previous studies have shown that milk production and milk composition in horses are influenced by breed, parity, lactation stage, management system, and environmental conditions [17,18,19]. In dairy horse breeds, milk yield generally increases with advancing parity, whereas fat and protein concentrations tend to decline during lactation [20,21]. Similar trends have been observed in other dairy species and are frequently explained by the dilution effect, whereby increasing milk volume is associated with lower concentrations of milk constituents [22,23,24]. Despite the growing number of studies on mare-milk composition, information regarding the relationships among milk yield, productive longevity, and lifetime productivity in native horse breeds remains limited.

Productive longevity is one of the most economically important traits in animal production because it directly affects lifetime productivity, replacement rates, and herd profitability. Studies in dairy cattle have demonstrated that first-lactation milk yield is strongly associated with subsequent productivity and survival [25,26]. Interestingly, maximum lifetime productivity is often achieved by animals with moderate-to-high rather than extremely high first-lactation milk yields. Excessive milk production during early lactations may increase physiological stress and reduce productive lifespan, whereas moderate production levels are frequently associated with greater longevity and higher cumulative lifetime yields [27,28]. Although these relationships have been extensively investigated in dairy cattle, comparable information is scarce for dairy horse breeds, including the Mugalzhar horse.

Growth performance during early life is another important indicator of future productive potential. Equine growth follows a characteristic sigmoid pattern, with rapid weight gain during the first months after birth followed by a gradual decline in growth rate as animals approach maturity [29]. Monitoring growth dynamics in colts and fillies provides valuable information for selection programs and breeding management. Furthermore, growth and morphometric evaluations are widely used in conservation and genetic-resource management programs for local horse breeds [30].

Despite the economic importance of the Mugalzhar horse, comprehensive studies simultaneously evaluating adult morphometric characteristics, foal growth and development, milk productivity, productive longevity, and milk composition remain limited. Moreover, the relationships among these traits have not been adequately explored using modern multivariate statistical approaches. Therefore, the objective of the present study was to evaluate the morphometric characteristics of adult Mugalzhar horses, analyze growth and development patterns in foals from birth to 18 months of age, determine the influence of first-lactation milk yield on productive longevity and lifetime productivity, assess changes in milk composition across successive lactations, and investigate relationships among productive and milk composition traits using principal component analysis, Pearson correlation analysis, and hierarchical cluster analysis.

## 2. Materials and Methods

### 2.1. Animals and Study Location

The study was conducted using Mugalzhar mares maintained under traditional pasture-based management conditions at the “Senim” farm located in the Suzak district of the Turkestan region, Kazakhstan. Records of milk production and productive longevity were obtained from the farm’s breeding and milk recording database.

A total of 120 adult Mugalzhar horses were used for morphometric evaluation, including 12 stallions and 108 mares. Productive longevity analysis was performed using historical production records from 100 mares that had completed their productive life and were culled from the dairy herd. Eight mares were excluded from the longevity analysis because health-related problems resulted in incomplete lifetime production records or premature removal from the study, preventing reliable assessment of productive longevity. Therefore, only mares with complete lifetime production records were included in the final longevity dataset. All animals originated from the same commercial Mugalzhar horse herd (“Senim” farm, Turkestan Region, Kazakhstan). Growth performance was evaluated in foals born in the same herd, and milk composition was determined from routine milk recording performed twice monthly during lactation. Repeated milk yield measurements were obtained from the same mares throughout each lactation, whereas morphometric measurements were recorded once for each animal.

### 2.2. Milk Yield Recording and Productive Longevity Assessment

Milk production of lactating mares was assessed using routine milk recording data. Control milkings were performed twice monthly throughout the lactation period. Daily milk yield was estimated according to the method proposed by Saigin [12].

To evaluate the influence of first-lactation milk yield on productive longevity and lifetime productivity, mares were classified into six groups according to milk yield during the first lactation:

- G1: ≤1000 kg;
- G2: 1001–2000 kg;
- G3: 2001–2500 kg;
- G4: 2501–3000 kg;
- G5: 3001–3500 kg;
- G6: >3501 kg.

For each group, first-lactation yield, peak milk yield, lifetime milk production, productive herd life, and number of lactations were determined.

The classification thresholds were selected to represent the natural distribution of first-lactation milk yield observed in the study population and to facilitate biologically meaningful comparisons among mares with low, moderate, and high milk production. The 1000 kg interval was chosen because it reflects commonly recognized production categories in practical horse breeding and provided an adequate number of animals within each group for statistical analysis while preserving the biological interpretation of productivity differences.

### 2.3. Milk Sampling and Physicochemical Analysis

Milk samples were collected from clinically healthy lactating Mugalzhar mares during the second to third month of lactation, when milk production is relatively stable. Sampling was performed during routine control milkings under hygienic conditions using sterile containers. Foals were not separated from their dams before milking. Instead, milk collection was carried out in the presence of the foal to stimulate the natural milk let-down reflex, which is a standard milking practice for dairy mares. Before sample collection, the first streams of milk were discarded to minimize external contamination.

The physicochemical quality of milk was evaluated using a Lactan 3 milk analyzer (Sibagropribor, Novosibirsk, Russia). The Lactan 3 analyzer was operated according to the manufacturer’s instructions and routinely calibrated using the manufacturer’s calibration procedures before sample analysis.

Fat, protein, lactose, density, and total solids were determined instrumentally using the Lactan 3 analyzer. Titratable acidity was determined according to standard dairy analytical methods and expressed in degrees Turner (°T). All analyses were performed in duplicate, and the average value was used for statistical analysis.

### 2.4. Morphometric Measurements

Morphometric evaluation of adult Mugalzhar horses included determination of live weight, height at withers, body length, chest girth, and cannon girth. Measurements were obtained using standard zootechnical methods and compared with the official breed standards.

Growth performance of foals was evaluated from birth to 18 months of age through periodic live-weight measurements. Growth dynamics were analyzed separately for colts and fillies.

### 2.5. Statistical Analysis

Statistical analyses were performed using JMP Pro 17 (SAS Institute Inc., Cary, NC, USA). Data are presented as mean ± standard error (SE). Differences among the six first-lactation milk yield groups (G1–G6) were evaluated using one-way analysis of variance (ANOVA), followed by Tukey’s honestly significant difference (HSD) post hoc test for multiple comparisons. Different superscript letters indicate statistically significant differences between group means (*p* < 0.05).

Differences among the six first-lactation milk yield groups were evaluated using one-way ANOVA:

$$Y_{ij}=\mu +\alpha_{i}+\varepsilon_{ij}$$

where $Y_{ij}$ is the observed value, μ is the overall mean, $\alpha_{i}$ is the fixed effect of the *i*th first-lactation milk yield group (G1–G6), and $\varepsilon_{ij}$ is the random error.

To investigate relationships among productive and milk composition traits, multivariate statistical analyses were performed using JMP Pro 17. Principal component analysis (PCA) was applied to identify the major sources of variation and to visualize associations among first-lactation milk yield, peak milk yield, lifetime milk yield, productive herd life (PHL), number of lactations, and milk composition traits. Pearson correlation analysis was conducted to quantify pairwise relationships among variables. Hierarchical cluster analysis (HCA) using Ward’s linkage method and Euclidean distance was performed to classify first-lactation yield groups according to their productive and compositional characteristics.

## 3. Results

### 3.1. Morphometric Characteristics of Adult Mugalzhar Horses

The morphometric characteristics of adult Mugalzhar horses are presented in Table 1. Clear sex-related differences were observed for all measured traits, with stallions exhibiting larger body dimensions and greater live weight than mares. The average live weight of stallions reached 491.3 ± 13.4 kg, exceeding that of mares (455.7 ± 8.2 kg) by approximately 7.8%. Similar differences were observed for the principal body measurements, including height at withers, body length, chest girth, and cannon girth.

Stallions attained an average height at withers of 145.1 ± 0.47 cm and a body length of 150.9 ± 0.52 cm, whereas the corresponding values for mares were 143.3 ± 0.51 cm and 149.1 ± 0.55 cm, respectively. Chest girth averaged 182.3 ± 0.61 cm in stallions and 180.1 ± 0.57 cm in mares, reflecting the well-developed body conformation characteristic of the breed. Likewise, cannon girth was greater in stallions (20.3 ± 0.13 cm) than in mares (19.3 ± 0.09 cm), indicating a stronger skeletal structure in male animals.

Comparison with the official breed standards demonstrated a high degree of conformity of the studied population to the established Mugalzhar horse phenotype. The measured values for both stallions and mares closely matched the standard parameters for live weight and body dimensions, with only minor deviations. These findings indicate that the breeding population has maintained the characteristic morphometric features of the Mugalzhar breed and exhibits a stable and well-defined exterior type suitable for productive horse breeding systems.

### 3.2. Growth and Development of Mugalzhar Foals

The dynamics of live weight gain in Mugalzhar foals from birth to 18 months of age are presented in Table 2. Both colts and fillies exhibited continuous growth throughout the observation period, reflecting the intensive development characteristic of the breed during the postnatal period.

At birth, colts weighed 42.6 ± 0.3 kg, while fillies averaged 39.3 ± 0.7 kg, indicating an initial sex-related difference of approximately 8.4%. During the first month of life, live weight nearly doubled in both sexes, reaching 82.3 ± 1.7 kg in colts and 78.6 ± 0.7 kg in fillies. By three months of age, body weight increased to 124.8 ± 2.2 kg and 122.3 ± 0.9 kg, respectively, demonstrating rapid early growth associated with the milk-feeding period.

The most pronounced differences between sexes became evident after six months of age. At this stage, colts reached a live weight of 175.3 ± 1.5 kg, exceeding that of fillies (158.7 ± 2.4 kg) by approximately 10.5%. Growth continued steadily throughout the subsequent developmental stages, with body weight increasing to 225.9 ± 1.6 kg in colts and 222.6 ± 1.7 kg in fillies at 12 months of age.

At 18 months, the average live weight reached 289.0 ± 4.7 kg in colts and 268.9 ± 5.8 kg in fillies. Relative to birth weight, live weight increased approximately 6.8-fold in colts and 6.9-fold in fillies, confirming the high growth potential of Mugalzhar horses during the rearing period. Although both sexes followed similar growth trajectories, colts maintained consistently greater body weight throughout development, reflecting the onset of sexual dimorphism and the greater growth intensity characteristic of male animals.

**Figure 1:**
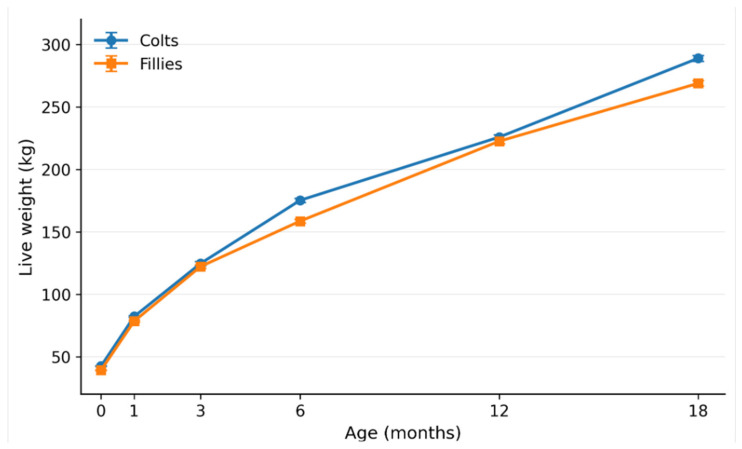
Growth curves of live weight in Mugalzhar colts and fillies from birth to 18 months of age. Values are presented as mean ± SE. Error bars represent the standard error of the mean.

The results indicate that Mugalzhar foals possess high growth performance and achieve substantial body weight gains during the first 18 months of life, supporting their suitability for productive horse breeding under the environmental conditions of Kazakhstan (Figure 1).

### 3.3. Effect of First-Lactation Milk Yield on Productive Longevity

The relationship between first-lactation milk yield and indicators of productive longevity is presented in Table 3. Significant differences (*p* < 0.05) were observed among the six production groups for all evaluated traits, indicating that the level of milk production during the first lactation was closely associated with subsequent productive performance and lifetime productivity.

As expected, first-lactation milk yield increased progressively from G1 to G6, ranging from 859.7 ± 71.2 kg in the lowest-producing group to 3705.5 ± 111.6 kg in the highest-producing group. A similar trend was observed for peak milk yield, which increased from 2638.2 ± 152.3 kg in G1 to more than 5000 kg in G4–G6. The highest peak yield was recorded in G4 (5200.3 ± 167.2 kg), although no significant differences were detected among the highest-producing groups (G4–G6).

Lifetime milk production did not increase linearly with first-lactation yield. The greatest lifetime yields were observed in the intermediate-to-high production groups G3 and G4, reaching 25,996.9 ± 995.1 kg and 24,961.0 ± 1200.2 kg, respectively. These values were significantly higher than those recorded in the remaining groups (*p* < 0.05). In contrast, despite their exceptionally high first-lactation yields, mares in G5 and G6 produced lower lifetime milk yields than those in G3 and G4.

A similar pattern was observed for productive longevity. The longest productive life was recorded in G3 (8.5 ± 0.8 years), followed by G4 (7.9 ± 0.9 years), whereas the shortest productive period was observed in G5 (4.8 ± 0.9 years). The number of completed lactations also differed significantly among groups, ranging from 3.1 ± 0.8 in G5 to 6.9 ± 0.7 in G3. Mares belonging to G2–G4 maintained significantly more lactations than those in G1, G5, and G6.

The results demonstrate that moderate-to-high milk production during the first lactation was associated with superior productive longevity and greater lifetime milk yield. In contrast, extremely high first-lactation productivity did not confer additional long-term advantages and was accompanied by a reduction in productive lifespan and the number of completed lactations. These findings suggest that optimal rather than maximal milk production during early lactations may provide the greatest long-term productive efficiency in Mugalzhar mares.

### 3.4. Changes in Milk Composition Across Lactations

Changes in milk yield and major milk components across successive lactations are presented in Table 4. Milk production varied considerably with lactation number, whereas the chemical composition of milk remained relatively stable throughout the productive period.

Milk yield increased progressively from the first to the fifth lactation. The average yield rose from 722.3 ± 19.3 kg during the first lactation to 986.9 ± 16.4 kg in the fifth lactation, representing an increase of approximately 36.6%. The highest milk production was therefore observed in mature mares during the middle productive period. Following this peak, milk yield declined markedly during the sixth lactation to 781.9 ± 28.1 kg, indicating a reduction in productive capacity with advancing age.

In contrast to milk yield, milk fat content exhibited a gradual decreasing trend from 1.76 ± 0.04% in the first lactation to 1.64 ± 0.02% in the fifth lactation. Although a slight increase to 1.73 ± 0.03% was observed during the sixth lactation, fat concentration remained within a relatively narrow range throughout the study period. Similar changes were recorded for protein content, which declined from 2.28 ± 0.03% in the first lactation to 2.17 ± 0.01% in the fourth lactation before showing a slight recovery in later lactations.

Lactose concentration was the most stable milk constituent, varying only between 6.87% and 7.12% across all lactations. The highest lactose content was recorded during the first lactation (7.12 ± 0.04%), while the lowest value was observed during the third lactation (6.87 ± 0.03%). Overall, fluctuations in lactose concentration were minimal compared with those observed for milk yield.

These results indicate that lactation number had a pronounced effect on milk productivity but only a moderate influence on milk composition. The increase in milk yield up to the fifth lactation suggests that maximum productive performance is achieved during the mature reproductive period, whereas the relatively stable concentrations of fat, protein, and lactose demonstrate the preservation of milk quality across successive lactations. The observed decline in milk yield after the fifth lactation may reflect age-related physiological changes affecting mammary gland productivity.

### 3.5. Multivariate Analysis of Productive Traits

**Figure 2:**
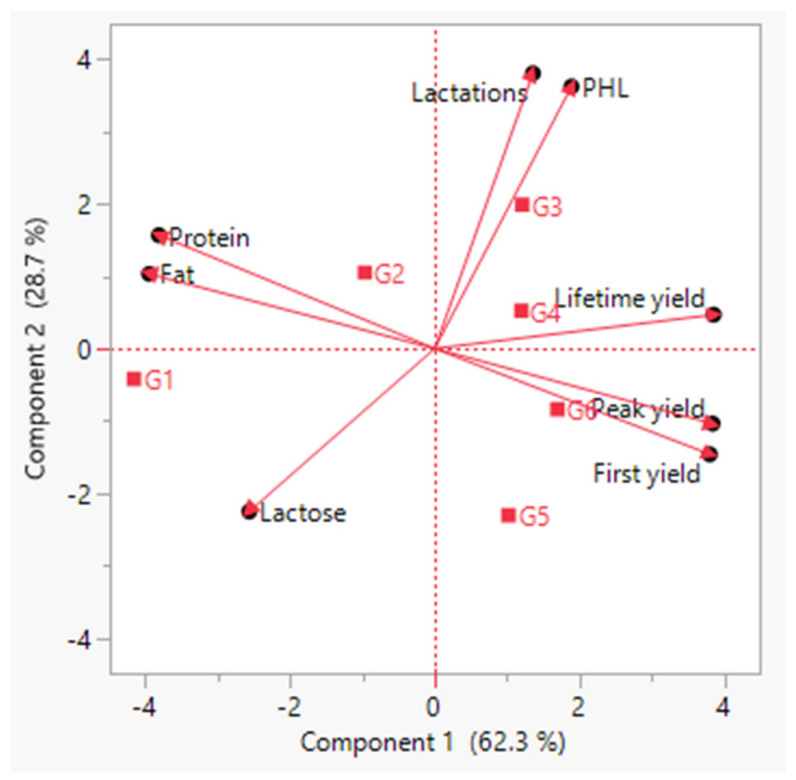
Principal Component Analysis of Productive and Milk Composition Traits.

PCA was performed to explore the relationships among productive longevity indicators and milk composition traits (Figure 2). The first two principal components explained 89.0% of the total variance, with PC1 accounting for 62.3% and PC2 accounting for 26.7%, indicating that the biplot provided an adequate representation of the multidimensional dataset.

The first PC1 was primarily associated with milk production traits. First-lactation yield, peak milk yield, and lifetime milk yield exhibited strong positive loadings on PC1 and clustered closely together on the positive side of the axis. This pattern indicates a strong positive relationship among these variables and demonstrates that animals with higher milk production during early lactations generally achieved greater cumulative lifetime productivity (Table S1).

Milk fat and protein contents were located on the negative side of PC1, opposite to the production-related variables. The opposite orientation of these vectors suggests an inverse relationship between milk quantity and the concentrations of fat and protein. Lactose also exhibited a negative loading on PC1 and was clearly separated from the major production traits, indicating a distinct pattern of variation compared with milk yield characteristics.

The second PC2 was largely influenced by productive longevity indicators, particularly the number of lactations and PHL, both of which showed strong positive loadings. The close proximity of these vectors indicates a strong positive association between productive lifespan and the number of completed lactations. Lifetime milk yield was positioned between the production-related variables and longevity traits, suggesting that cumulative productivity is influenced by both milk yield potential and the duration of productive use.

The distribution of production groups further highlighted substantial differences in performance profiles. Groups G3 and G4 were located in the positive region of both PC1 and PC2, indicating a favorable combination of high milk production and extended productive longevity. In particular, G3 was closely associated with productive life and number of lactations, reflecting its superior lifetime performance. Group G4 was positioned nearer to lifetime milk yield, suggesting efficient long-term productivity (Table S2).

Groups G5 and G6 were associated primarily with first-lactation and peak milk yield but were separated from the longevity variables. This distribution suggests that exceptionally high first-lactation milk yield was not accompanied by a proportional increase in productive lifespan. Conversely, G1 occupied the negative region of PC1 and was clearly separated from the remaining groups, reflecting its comparatively low productivity and limited lifetime performance. Group G2 showed an intermediate position between G1 and the higher-producing groups, indicating moderate productive characteristics.

PCA revealed two major biological gradients within the population: a productivity axis represented by milk yield traits and a longevity axis represented by productive life and number of lactations. The results demonstrate that intermediate-to-high producing mares (G3–G4) achieved the most favorable balance between milk production and productive longevity, whereas extremely high first-lactation milk yield (G5–G6) was not associated with superior lifetime performance.

**Figure 3:**
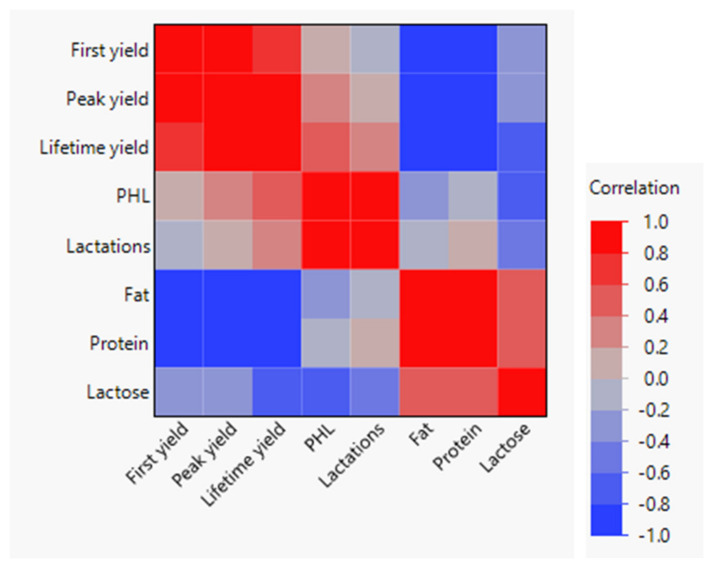
Pearson Correlation Matrix of Productive and Milk Composition Traits.

The Pearson correlation matrix (Figure 3) was used to evaluate the relationships among milk production traits, productive longevity indicators, and milk composition parameters. Distinct positive and negative correlation patterns were observed, indicating complex interactions between productivity, longevity, and milk quality traits.

Strong positive correlations were identified among first-lactation milk yield, peak milk yield, and lifetime milk yield, as evidenced by the intense red coloration within this cluster. These relationships indicate that mares producing larger quantities of milk during early lactations generally maintained higher production levels throughout life and achieved greater cumulative milk yield. The close association among these traits supports the importance of first-lactation milk yield performance as a predictor of long-term productivity.

PHL and the number of lactations also exhibited strong positive correlations with lifetime milk yield and moderate positive correlations with first-lactation and peak milk yields. This pattern suggests that extended productive longevity contributes substantially to cumulative milk production. The particularly strong association between PHL and the number of lactations reflects the biological dependence of lifetime productivity on the duration of productive use within the herd.

Milk fat and protein contents formed a separate positively correlated cluster. The strong positive relationship between these two variables indicates that increases in milk fat concentration were generally accompanied by corresponding increases in protein content. This finding is consistent with the coordinated synthesis of milk solids during lactation.

Notably, milk fat and protein concentrations exhibited strong negative correlations with first-lactation yield, peak yield, and lifetime yield, as shown by the pronounced blue coloration in the correlation matrix. These inverse relationships suggest a trade-off between milk quantity and milk quality, whereby animals producing larger volumes of milk tended to have lower concentrations of fat and protein. Such dilution effects are commonly observed in high-producing dairy animals.

Lactose demonstrated a distinct correlation pattern compared with the other milk components. Although lactose was positively associated with fat and protein content, it showed weak-to-moderate negative correlations with the major milk yield traits. The relatively lower magnitude of these correlations indicates that lactose concentration remained comparatively stable across different production levels.

The correlation analysis identified two major groups of traits: production and longevity indicators, including first-lactation yield, peak yield, lifetime yield, productive herd life, and number of lactations, which were positively interrelated; and milk composition traits, particularly fat and protein content, which were negatively associated with milk yield characteristics. These findings support the PCA results and further demonstrate that maximum lifetime productivity in Mugalzhar mares is determined not only by milk yield potential but also by productive longevity, whereas elevated milk production is generally accompanied by lower concentrations of milk solids.

**Figure 4:**
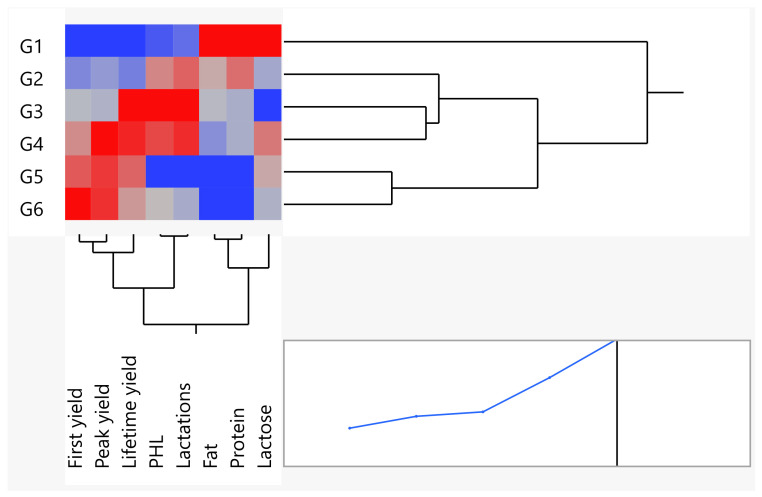
Hierarchical Cluster Analysis of First-Lactation Yield Groups.

HCA was performed to further evaluate similarities among the six first-lactation milk yield groups based on productive longevity and milk composition traits (Figure 4). The resulting dendrogram revealed a clear separation of the groups into distinct clusters, reflecting differences in their production profiles and lifetime performance.

The closest similarity was observed between groups G3 and G4, which clustered together at the shortest linkage distance. These groups were characterized by the highest lifetime milk yields, extended productive lifespan, and a greater number of completed lactations. Their close association indicates that mares with moderate-to-high first-lactation milk production achieved the most favorable balance between milk productivity and productive longevity.

Group G2 clustered with G3 and G4 at the next hierarchical level, forming a larger cluster of animals characterized by relatively long productive lives and above-average lifetime milk production. Although the first-lactation yield of G2 was lower than that of G3 and G4, its productive lifespan and number of lactations were comparable, indicating a similar long-term production strategy.

A second major cluster consisted of groups G5 and G6, which were closely associated with one another. These groups were characterized by exceptionally high first-lactation and peak milk yields but relatively lower productive longevity and fewer completed lactations. The clustering pattern suggests that extremely high first-lactation milk yield was associated with a different biological profile than that observed in the more balanced G2–G4 groups.

Group G1 formed a distinct branch and was clearly separated from all other groups. This group was characterized by the lowest values for first-lactation yield, peak yield, lifetime milk production, productive herd life, and number of lactations. The isolated position of G1 reflects its substantially lower productive performance compared with the remaining groups.

The accompanying heatmap supports the dendrogram structure and illustrates the distribution of trait values across groups. High-producing and long-lived groups (G3 and G4) exhibited elevated values for lifetime yield, productive herd life, and number of lactations, whereas G5 and G6 were primarily associated with high first-lactation and peak milk yields. Conversely, G1 showed low values for most production-related traits but relatively higher concentrations of milk composition traits, particularly fat and protein content.

The hierarchical clustering analysis identified three biologically meaningful production patterns within the population: low-producing animals with limited productive longevity (G1), intermediate-to-high producing mares with superior lifetime productivity and longevity (G2–G4), and very high early-producing animals characterized by elevated milk yield but reduced productive lifespan (G5–G6). These findings corroborate the PCA and correlation analyses and demonstrate that the most efficient production strategy in Mugalzhar mares is associated with balanced milk yield and extended productive longevity rather than maximal early-lactation production.

## 4. Discussion

The present study provides an integrated assessment of morphometric development, growth performance, productive longevity, and milk composition in the Mugalzhar horse, a dual-purpose native breed of Kazakhstan. By combining classical zootechnical evaluation with multivariate statistical approaches, the results offer new insight into how early-life productivity relates to lifetime performance and milk quality in this breed, a relationship that has previously been characterized mainly in dairy cattle but has remained largely unexplored in native horse populations.

The morphometric measurements obtained for adult stallions and mares closely matched the official Mugalzhar breed standard, with stallions exceeding mares by approximately 7.8% in live weight and showing consistently larger linear body measurements. This pattern of sexual dimorphism in body size is typical of equine breeds and is consistent with previous zootechnic characterizations of Mugalzhar horse populations reporting comparable body dimensions and a stable, well-defined exterior type [3]. The close agreement between the measured values and the breed standard suggests that the “Senim” farm population has preserved the morphological integrity of the breed despite being managed under extensive, pasture-based conditions, supporting the broader conclusion that phenotypic monitoring is an effective and low-cost tool for tracking conservation status in local breeds [31,32]. These observations are consistent with previous studies reporting that the Mugalzhar horse has maintained stable morphometric characteristics and close conformity to the official breed standard despite long-term selection under extensive grazing conditions [8,9,10]. Maintaining this conformity is particularly relevant given that genetic studies have documented considerable diversity and distinct adaptive signatures within the Mugalzhar breed [33], indicating that phenotypic stability and underlying genetic variability are not mutually exclusive and that selection in this population has so far not eroded the standard body type.

Growth performance from birth to 18 months followed the sigmoid trajectory typical of equine postnatal development, with the most rapid relative gains occurring during the first three months of life, coinciding with the period of highest milk dependency, followed by a progressive decline in growth rate as foals approached yearling age [29,34]. The approximately 6.8- to 6.9-fold increase in live weight observed between birth and 18 months in both sexes indicates that Mugalzhar foals achieve substantial early growth under extensive pasture management, without reliance on intensive supplementary feeding [35]. Sex-related divergence became most pronounced after six months of age, when colts exceeded fillies by roughly 10.5%, a pattern consistent with the earlier onset of sexual dimorphism reported for other horse breeds [30] and with general patterns of growth and development described for the genus Equus [36]. From a practical standpoint, the consistency of these growth curves with breed-standard expectations supports their use as a benchmark for selection and management decisions, and reinforces the value of growth monitoring as a component of genetic-resource conservation programs for native horse breeds [37]. The growth pattern observed in the present study is also consistent with previous reports demonstrating that the most intensive postnatal growth in horses occurs during the first months of life under extensive pasture-based management [29,30].

A central finding of this study is that the relationship between first-lactation milk yield and lifetime productivity in Mugalzhar mares is non-linear. Although first-lactation and peak yields increased progressively from G1 to G6, lifetime milk production and productive herd life peaked in the intermediate-to-high groups G3 and G4, while the highest-yielding groups, G5 and G6, achieved shorter productive lifespans and fewer completed lactations despite their elevated early production. This pattern is consistent with observations reported in dairy cattle, where animals with moderate-to-high first-lactation milk yield often achieve greater lifetime productivity than those with extremely high early production. [25]. However, because the present study was based on retrospective observational data, these findings should be interpreted as associations rather than evidence of causal relationships. Other factors, including genetic background, health status, reproductive performance, nutritional management, and culling decisions, may also have contributed to the observed differences among groups. Therefore, further prospective longitudinal studies are needed to clarify the biological mechanisms underlying these associations [27]. The present results extend this principle, established primarily in Holstein and other dairy cattle populations [38], to a native dairy horse breed managed under extensive pasture conditions, suggesting that the trade-off between early productivity and longevity may be a broadly conserved feature of dairy animal physiology rather than one specific to intensively managed cattle systems. The relatively stable concentrations of fat, protein, and lactose across successive lactations are consistent with previous reports indicating that lactation number has a greater influence on milk yield than on the major compositional characteristics of mare’s milk [17,18,19,20,21].

These findings suggest that moderate-to-high first-lactation milk yield may represent a favorable production profile in the studied population. Nevertheless, selection decisions should not rely solely on first-lactation milk yield, because productive longevity is influenced by multiple biological and management factors. Future breeding programs should therefore evaluate first-lactation milk yield together with health, reproductive performance, and longevity traits before implementing selection strategies [39,40]. This is consistent with general livestock-breeding theory in which functional longevity is treated as a trait with direct economic value distinct from, and sometimes inversely related to, peak production [41]. Our results are consistent with recent observations in dairy cattle, suggesting that first-lactation performance is an important predictor of lifetime productivity. Fahim et al. [42] reported that Holstein cows with higher first-lactation milk yield achieved greater lifetime milk production, although this advantage was accompanied by reduced reproductive performance.

Milk yield increased progressively from the first to the fifth lactation, by approximately 36.6%, before declining in the sixth lactation, while fat and protein concentrations showed a gradual decline over the same period before recovering slightly in later lactations, and lactose remained the most stable constituent throughout. This trajectory, rising yield with advancing parity up to a point of maximum maturity followed by a decline, is broadly consistent with patterns described for other dairy species and with the general principle that milk yield and lactation stage jointly shape milk composition [20,43]. The inverse relationship observed between milk volume and fat or protein concentration is consistent with the dilution effect described in dairy cattle and other livestock, whereby increases in secreted milk volume are accompanied by a relative decrease in the concentration of milk solids rather than a true reduction in total solids output [44,45]. The comparatively high stability of lactose across lactations mirrors its role as the principal osmotic regulator of milk volume in mammals and is consistent with previous reports describing mare’s milk as compositionally distinct from bovine milk in having proportionally higher lactose and lower fat content, a feature that contributes to its similarity with human milk and its suitability for koumiss production and other functional dairy applications [46,47]. The decline in yield after the fifth lactation likely reflects age-related reductions in mammary secretory capacity, a phenomenon also documented in other equine populations under pasture-based management. These findings are consistent with recent evidence indicating that mare’s milk possesses unique nutritional and functional characteristics that extend beyond its basic chemical composition. A recent comprehensive review by Shokrollahi et al. [48] highlighted that mare’s milk is characterized by an approximately 1:1 casein-to-whey protein ratio, high concentrations of lysozyme, lactoferrin, and immunoglobulins, and the generation of more than 2300 bioactive peptides during koumiss fermentation. The relatively small differences in the concentrations of major milk constituents among milk yield groups may reflect the well-recognized dilution effect associated with increased milk production. Craig et al. [49] reported that high-producing dairy cows tended to produce milk with lower fat and protein concentrations, which was attributed to a combination of genetic factors, nutritional management, and the dilution effect resulting from increased milk volume.

Several limitations should be considered when interpreting these findings. The study was conducted at a single farm under one extensive pasture-management regime, so the generalizability of the productivity and longevity relationships to Mugalzhar populations managed under different systems, feeding regimes, or climatic conditions remains to be confirmed. The retrospective nature of the longevity dataset, comprising mares that had already completed their productive life, may also be subject to survivorship-related bias. Furthermore, detailed individual records on reproductive performance, health status, and reasons for culling were not available for all mares and therefore could not be included in the statistical analyses. Likewise, potential confounding effects related to year, season, parity, and management practices were not explicitly evaluated and may have influenced the observed associations. Future studies incorporating genomic data, such as the whole-genome resources already available for this breed, could help clarify whether the productivity–longevity trade-off identified here has an identifiable genetic basis, which would allow it to be incorporated directly into marker-assisted selection programs. Longitudinal, multi-farm studies that track individual mares from birth through their entire productive life, combined with detailed nutritional and reproductive records, would further help disentangle the relative contributions of genetics, management, and physiological trade-offs to the patterns observed. Finally, extending the milk-composition analysis to include minor bioactive components, such as lactoferrin, lysozyme, and immunoglobulins, which have been highlighted as nutritionally and therapeutically important in mare’s milk, would provide a more complete picture of how lactation number and selection for yield affect the functional, and not only the macronutrient, quality of Mugalzhar mare’s milk.

## 5. Conclusions

This study demonstrated that Mugalzhar horses exhibited good growth performance, stable milk composition across lactations, and productive characteristics consistent with breed standards. Mares with moderate first-lactation milk yield showed the greatest lifetime milk production and longest productive herd life, whereas extremely high first-lactation milk yield was not associated with superior lifetime performance. These findings suggest that first-lactation milk yield may be considered together with longevity and other functional traits in future breeding programs. The integrated evaluation of growth performance, milk productivity, productive longevity, and milk composition provides a comprehensive phenotypic characterization of the Mugalzhar breed under extensive pasture-based management conditions. The results contribute to a better understanding of the relationships among productive traits in this important native horse breed and provide a useful reference for future breeding and conservation programs. Because this study was based on a single herd and a retrospective observational design, the findings should be interpreted as associations rather than causal relationships. Further multicenter longitudinal studies incorporating reproductive, health, nutritional, and genomic information are warranted to validate and extend the present findings.

The findings of this study provide practical information for breeding and management programs aimed at improving the productivity and sustainability of the Mugalzhar horse breed. The identified relationships between first-lactation milk yield, lifetime productivity, and productive longevity may assist breeders in selecting mares with superior long-term performance while maintaining herd health and reproductive efficiency. Furthermore, these results contribute to the conservation and sustainable utilization of this indigenous genetic resource by providing scientifically based selection criteria for breeding programs in Kazakhstan.

## Figures and Tables

**Table 1:** Body measurements and live weight of Mugalzhar horses.

Trait	Stallions (*n* = 12)	Mares (*n* = 108)	Breed Standard
Live weight (kg)	491.3 ± 13.4	455.7 ± 8.2	480/460
Height at withers (cm)	145.1 ± 0.47	143.3 ± 0.51	145/143
Body length (cm)	150.9 ± 0.52	149.1 ± 0.55	151/149
Chest girth (cm)	182.3 ± 0.61	180.1 ± 0.57	182/180
Cannon girth (cm)	20.3 ± 0.13	19.3 ± 0.09	19.5/19.0

**Table 2:** Growth and development of Mugalzhar foals.

Age (Months)	Live Weight, Colts (kg)	Live Weight, Fillies (kg)
Birth	42.6 ± 0.3	39.3 ± 0.7
1	82.3 ± 1.7	78.6 ± 0.7
3	124.8 ± 2.2	122.3 ± 0.9
6	175.3 ± 1.5	158.7 ± 2.4
12	225.9 ± 1.6	222.6 ± 1.7
18	289.0 ± 4.7	268.9 ± 5.8

**Table 3:** Effect of first-lactation milk yield on productive longevity.

Group	*n*	First-Lactation Yield (kg)	Peak Yield (kg)	Lifetime Yield (kg)	Productive Life (Years)	Number of Lactations
G1	10	859.7 ± 71.2 ^d^	2638.2 ± 152.3 ^d^	6103.9 ± 791.4 ^d^	5.2 ± 0.8 ^b^	3.9 ± 0.7 ^b^
G2	25	1750.1 ± 86.7 ^c^	3800.1 ± 181.6 ^c^	12,425.7 ± 649.8 ^c^	7.3 ± 0.7 ^a^	6.1 ± 0.7 ^a^
G3	23	2359.2 ± 92.1 ^b^	4111.7 ± 116.8 ^b^	25,996.9 ± 995.1 ^a^	8.5 ± 0.8 ^a^	6.9 ± 0.7 ^a^
G4	22	2804.6 ± 76.4 ^b^	5200.3 ± 167.2 ^a^	24,961.0 ± 1200.2 ^a^	7.9 ± 0.9 ^a^	6.6 ± 0.7 ^a^
G5	10	3150.3 ± 99.5 ^a^	4965.9 ± 122.1 ^a^	22,367.1 ± 1376.3 ^b^	4.8 ± 0.9 ^b^	3.1 ± 0.8 ^b^
G6	10	3705.5 ± 111.6 ^a^	5006.6 ± 172.4 ^a^	20,309.1 ± 887.9 ^b^	6.8 ± 0.8 ^a^	4.9 ± 0.9 ^b^

Values are presented as mean ± SE. Different superscript letters indicate significant differences among groups according to one-way ANOVA followed by Tukey’s HSD test (*p* < 0.05).

**Table 4:** Changes in milk composition across lactations.

Lactation	Milk Yield (kg)	Fat (%)	Protein (%)	Lactose (%)
1	722.3 ± 19.3	1.76 ± 0.04	2.28 ± 0.03	7.12 ± 0.04
2	783.8 ± 22.1	1.75 ± 0.01	2.26 ± 0.04	6.98 ± 0.03
3	829.1 ± 28.5	1.73 ± 0.01	2.23 ± 0.01	6.87 ± 0.03
4	943.5 ± 18.2	1.68 ± 0.03	2.17 ± 0.01	7.05 ± 0.02
5	986.9 ± 16.4	1.64 ± 0.02	2.19 ± 0.02	7.02 ± 0.03
6	781.9 ± 28.1	1.73 ± 0.03	2.21 ± 0.01	6.99 ± 0.03

## Data Availability

The original contributions presented in this study are included in the article/Supplementary Material. Further inquiries can be directed to the corresponding authors.

## Supplementary Materials

The following supporting information can be downloaded at: https://www.mdpi.com/article/10.3390/ani16142272/s1. Table S1: PCA loading matrix; Table S2: Pearson correlation coefficients.

## Abbreviations

The following abbreviations are used in this manuscript:

HCA	Hierarchical Cluster Analysis
PCA	Principal Component Analysis
PHL	Productive Herd Life
ANOVA	Analysis of Variance
HSD	Tukey’s honestly significant difference test
