# Supplementary material for: Growth Performance, Milk Productivity, Productive Longevity, and Milk Composition of Mugalzhar Horses
Source: Animals (Basel). 2026 Jul 22;16(14):2272. doi: 10.3390/ani16142272 (PMC13405355; doi:10.3390/ani16142272)
Supplement: Supplementary file 1 [file animals-16-02272-s001.zip › animals-4420848-supplementary.pdf]

Supplementary Table S1. PCA loading matrix.

	Prin1	Prin2
First yield	0.91403	-0.35143
Peak yield	0.92330	-0.24946
Lifetime yield	0.92710	0.11287
PHL	0.45436	0.87150
Lactations	0.32641	0.91599
Fat	-0.94957	0.24858
Protein	-0.91729	0.37740
Lactose	-0.61670	-0.54184

Supplementary Table S2. Pearson correlation coefficients.

	First yield	Peak yield	Lifetime yield	PHL	Lactations	Fat	Protein	Lactose
First yield	1.0000	0.9355	0.7643	0.1361	-0.0082	-0.9519	-0.9754	-0.3427
Peak yield	0.9355	1.0000	0.8320	0.2411	0.1386	-0.9429	-0.9051	-0.2782
Lifetime yield	0.7643	0.8320	1.0000	0.5014	0.3938	-0.8023	-0.8027	-0.6036
PHL	0.1361	0.2411	0.5014	1.0000	0.9841	-0.2100	-0.0753	-0.6626
Lactations	-0.0082	0.1386	0.3938	0.9841	1.0000	-0.0858	0.0723	-0.5716
Fat	-0.9519	-0.9429	-0.8023	-0.2100	-0.0858	1.0000	0.9606	0.4766
Protein	-0.9754	-0.9051	-0.8027	-0.0753	0.0723	0.9606	1.0000	0.4219
Lactose	-0.3427	-0.2782	-0.6036	-0.6626	-0.5716	0.4766	0.4219	1.0000
